# List of Reviewers

**DOI:** 10.1002/pcn5.56

**Published:** 2022-12-08

The Editorial Board members of Psychiatry and Clinical Neurosciences Reports are grateful to the following reviewers who provided the Journal with their expertise from 27^th^ October 2021 to 30^th^ September 2022. Their efforts are greatly appreciated.

**Table:** 

Anton, Steve
Asai, Mariko
Asherson, Philip
Ayani, Nobutaka
Baba, Hajime
Boku, Shuken
Chen, Chong
Farokhnezhad Afshar, Pouya
Ferrando, Stephen
Fujino, Junya
Fukase, Yuko
Funatogawa, Tomoyuki
Fushimi, Masahito
Gaetz, William
Hashimoto, Naoki
Hori, Hikaru
Iga, Junichi
Ikebuchi, Emi
Ikenouchi, Atsuko
Ikuta, Toshikazu
Imai, Atsushi
Imai, Hissei
Imamura, Akira
Imamura, Yayoi
Inoue, Keisuke
Inoue, Shinichiro
Inoue, Takeshi
Ishida, Yasushi
Isobe, Masanori
Ito, Hiroto
Ito, Masaya
Iwamoto, Kazuya
Iwamoto, Kunihiro
Iwanami, Akira
Iwata, Masaaki
Kanazawa, Tetsufumi
Kato, Masaki
Kawabe, Kentaro
Kawashima, Yoshitaka
Kishi, Yasuhiro
Kishimoto, Toshifumi
Kliem, Soren
Ko, Chih‐Hung
Kobayashi, Ryota
Kosaka, Hirotaka
Kozuki, Haruka
Kuroki, Noriomi
Kyriakopoulos, Marinos
Landi, Giulia
Lin, Chung‐Ying
Matsuda, Yuki
Matsumoto, Takuya
Matsuo, Koji
Matsuoka, Teruyuki
Metoki, Hirohito
Miyawaki, Dai
Moriguchi, Sho
Mortazavi, Forough
Murayama, Keitaro
Myers, Lorna
Nagamine, Takahiko
Nakajima, Madoka
Nakamura, Katsunori
Nakamura, Masayuki
Nakashima, Yoshifumi
Narumoto, Jin
Naz, Marzieh Saei Ghare
Niimura, Hidehito
Nishida, Keiichiro
Nishigori, Hidekazu
Noda, Takamasa
Noda, Yoshihiro
Noguchi, Masayuki
Ochi, Taichi
Oda, Hiroyuki
Ogawa, Asao
Ogawa, Shintaro
Ohi, Kazutaka
Ojio, Yasutaka
Okada, Takashi
Okondo Sinsuke
Okubo, Ryo
Okumura, Yasuyuki
Ootsu, Hiroshi
Oribe, Naoya
Ota, Toyosaku
Philipsen, Alexandra
Pompili, Maurizio
Sadahiro, Ryoichi
Saito, Junichi
Saito, Takuya
Sakamoto, Shinji
Sasaki, Megumi
Sasaki, Tsuyoshi
Sato, Takeshi
Serafini, Gianluca
Sherrard, R. M.
Shimada, Hiroyuki
Shimizu, Eiji
Shimoda, Kengo
Shinagawa, Shunichiro
Shinohara, Kiyomi
Shoji, Mikio
Squassina, Alessio
Suzuki, Futoshi
Tachikawa, Hirokazu
Takaesu, Yoshikazu
Takahashi, Hidetoshi
Takahashi, Shun
Takechi, Hajime
Takekita, Yoshiteru
Takeshima, Tadashi
Tamune, Hidetaka
Tanaka, Shinichiro
Tani, Hideaki
Tateno, Masaru
Tor, Phern‐Chern
Tseng, Mei‐Chih M.
Tsermpini, Evangelia Eirini
Tsujii, Noa
Tsuneoka, Toshiaki
Ueno, Fumihiko
Usami, Masahide
Vesnaver, Elisabeth
Wada, Ken
Watanabe, Anri
Watanabe, Koichiro
Wolkowitz, Owen
Xiao, Gelei
Yagyu, Kazuyori
Yamaguchi, Sosei
Yamanaka, Katsuo
Yamashita, Hiroshi
Yasui‐Furukori, Noiro
Yonemoto, Naohiro
Yoshimura, Masafumi
Yoshimura, Reiji
Yoshinaga, Naoki

